# Knowledge Sharing Maturity Model for Medical Imaging Departments: Development Study

**DOI:** 10.2196/54484

**Published:** 2025-05-06

**Authors:** Maryam Almashmoum, James Cunningham, John Ainsworth

**Affiliations:** 1 Division of Informatics Imaging and Data Sciences School of Health Sciences Faculty of Biology, Medicine, and Health University of Manchester Manchester United Kingdom; 2 Nuclear Medicine Department Faisal Sultan Bin Eissa Kuwait Cancer Control Center Kuwait city Kuwait

**Keywords:** knowledge management, knowledge sharing, medical imaging departments, cancer centers, the Christie, Kuwait Cancer Control Center, KCCC, maturity model, factors, indicators, measurement

## Abstract

**Background:**

Knowledge sharing in medical imaging departments is driven by the need to improve health care services, develop health care professionals’ skills, and reduce repetitive mistakes. It is considered an important step in the implementation of knowledge management solutions. By following a maturity model of knowledge sharing, knowledge-sharing practices can be improved.

**Objective:**

This study aimed to develop a maturity model for knowledge sharing in the medical imaging department to help managers to assess the level of maturity of knowledge-sharing practices. In modern health care institutions, improvements in health care professionals’ skills and health care services are often driven through practicing knowledge-sharing behaviors. Managers can follow the indicators of maturity model of knowledge sharing and its measurements to identify the current level and move to the next level.

**Methods:**

This study was conducted in 4 stages: an *overview stage* that highlighted the factors that affect knowledge-sharing practices in medical imaging departments; an *analysis factor stage* that was designed to assess the factors that affect knowledge sharing using a concurrent mixed methods approach (questionnaires and semistructured interviews) in 2 medical imaging departments; *a structuring maturity model knowledge sharing stage*, where a maturity model of knowledge sharing was developed based on the findings of the first and second stages; and finally, an *assessment of reliability and validity stage*, where a modified Delphi method was used to obtain consensus among experts on model components to be ready for implantation.

**Results:**

The model presented in this study includes 17 indicators, divided into 11 components. Those components were derived from the findings of the questionnaires and semistructured interviews that were applied in the medical imaging departments. It consisted of 5 maturity levels: initial, aware, defined, managed, and optimized. In each level, measurements were included to help managers assess the current level by answering the questions. On the basis of reliability, the experts reached a consensus agreement on the model’s components in 2 rounds with SD <1.

**Conclusions:**

This maturity model of knowledge sharing in medical imaging departments allows managers and policy makers to measure the maturity level of knowledge sharing in those departments. Although the model has been applied to medical imaging departments, it could easily be modified for application in other institutions.

## Introduction

### Background

Knowledge is an essential asset in achieving successful institutional practices, and sharing knowledge gives a sustainable competitive advantage to modern institutions [1,2]. Accessing knowledge is the main step in problem-solving and decision-making [3]. Knowledge is a mixture of experiences, thoughts, ideas, values, and information. In the context of institutions, knowledge can be shared and transformed among employees within institutions to create new experiences and information that did not exist before [4]. It consists of 2 main categories: tacit and explicit knowledge [5]. Tacit knowledge is any knowledge that exists in human minds, such as thoughts and ideas. This kind of knowledge is difficult to document and share. In contrast, explicit knowledge is the knowledge that exists in policies, letters, documents, and manuals. This kind of knowledge is easy to document and share with others using several mechanisms [6]. Both kinds of knowledge are not separate from each other. The dynamic process between tacit and explicit knowledge helps share knowledge efficiently [5,7].

Health care institutions, for example, hospitals and specialized centers, must apply a knowledge management system to build a proper and effective network among all health care providers [4]. The reasons for implementing a knowledge management system are mainly the complexity and huge number of knowledge-based resources that need to be managed [8]. Implementing knowledge management depends on understanding its processes (knowledge creation, knowledge capture, knowledge sharing, and knowledge applications). Moreover, identifying a clear framework to follow is vital to targeting any weaknesses in each step and creating a good environment for health care professionals. As a result, health care services and patient outcomes can be improved [8].

Knowledge sharing is considered an important step in implementing knowledge management. Without a knowledge-sharing process, knowledge management will be difficult to implement because it relies on the dynamic process of tacit and explicit knowledge sharing among employees. Therefore, creating a culture of knowledge sharing among them, and understanding the resources and factors that affect knowledge sharing, are important for any knowledge management system. Looking at health care services, knowledge sharing is an essential step for successful knowledge management within institutions, as it can lead to improved health care settings performance, save them time, reduce costs, and increase health education levels [9]. It plays an important role in providing great accountability and establishing good practices in health planning and policy making. Abzari et al [10] stated that knowledge sharing is considered an essential factor in successful institutional performance. It refers to the act of sharing both tacit and explicit knowledge, such as thoughts, ideas, and experiences from one person, group, or institution to another to formulate new knowledge [11]. Knowledge sharing is one of the challenges for health care institutions due to the variety of resources and knowledge. Health care institutions are complex environments given the variety of specialties in each department and who operates institutional resources in each department [12].

Medical imaging departments are essential in any health care institution due to the importance of performing important procedures for the pretreatment plan and interpreting results. On the basis of the observation at the cancer center, there are several problems facing knowledge-sharing practices in the medical imaging department, such as a lack of awareness of the importance of knowledge sharing, difficulty in sharing knowledge among health care professionals, and a lack of implementation of information and communication technology (ICT) [13]. Creating a communication environment is important to control the amount of knowledge shared among health care professionals in their institutions [14]. In addition, dealing with health care institutional issues is directly related to human capital [15]. Sharing knowledge among health care professionals is critical to apply that knowledge in their daily work and allow them to create new knowledge that is needed for developing the institutional process. Both tacit and explicit knowledge are important to enhance the knowledge-sharing process among health care professionals, because they are directly related to the health care professional’s experience and skills [16]. Therefore, identifying a clear maturity model (MM) for knowledge sharing in medical imaging is important to help policy makers and managers use it to enhance knowledge-sharing practices among health care professionals.

### An Overview of the MMs

Maturity in this context refers to the degree to which technology, institutional process, or frameworks evolve over time [17,18]. In institutions, an MM can be methodically used to define operations and identify stages, which can lead to policy plans [19]. The concept of the MM was developed in the early 1970s [20]. It is increasingly applied in information systems [21] and is established in many fields, such as knowledge management, information management, and software performance and management [22-25]. The purpose of MMs is to provide a clear model based on an institution’s capabilities in a certain managerial area using a set of criteria and related evaluation methodologies [26]. It can be a powerful tool that helps identify strengths and weaknesses in a specific area [27]. Bititci et al [28] indicated that MMs have a positive impact on improving institutional performance and respond to many challenges by describing each step and stage properly.

Health care institutions face challenges in achieving best practices in knowledge management system implementation [29]. Maturity in knowledge management is defined as the degree to which knowledge assets are effectively managed and controlled within institutions [30]. There have been several MMs for evaluating and describing these areas of management that have been proposed in different studies [17-19,31-33]. Some other applications of MMs have been applied to health care intuition information systems [34].

On the basis of the definition of maturity in knowledge management, there is a strong link with the knowledge-sharing definition, which is sharing experience and knowledge with each other within an institution. This knowledge, either tacit or explicit, needs to be managed to be able to be shared. In addition, to share any kind of knowledge, it should be managed by human experience; this kind of management is required for implementing the knowledge management process because it is *dynamic and active* [35]. Therefore, there is a strong relationship between the 2 definitions, as both deal with the assets that need to be managed to obtain the objective. To share the knowledge, they must understand the types of knowledge and the factors that control the process of knowledge sharing, all of which need to be managed and controlled within institutions. On the basis of this context, to improve the knowledge management process, we need to improve the process of sharing the knowledge.

There is a continuing need to structure and develop a new knowledge sharing MM (KSMM) for enhancing knowledge-sharing practices among employees in the medical imaging departments [36]. Structuring a KSMM helps decision makers to achieve institutional tasks and improve the quality of patient outcomes [37]. There are several models of knowledge sharing [38-41]. Despite the variety of these models, few of them focus on knowledge sharing in health care in general and in the medical imaging department in particular [42]. There are several factors that affect knowledge sharing among health care professionals, including the MM of multidisciplinary team (MDT) meetings, community of practice, ICT, social media networks, picture archiving and communication system (PACS), telemedicine, and digital library [43-49]. Creating a MM for knowledge sharing that consists of all factors that affect their behaviors is vital to implementing a knowledge-sharing environment among health care professionals. The KSMM should be an umbrella that covers all the factors.

To date, there is no MM to assess knowledge-sharing practices among health care professionals in general hospitals, or in medical imaging departments specifically, covering all the factors that affect knowledge sharing in medical imaging departments. Ipe [50] represented the model for the relation of the knowledge sharing between individuals, factors, and the relation between them. Therefore, developing an MM to evaluate the level of maturity of knowledge-sharing behaviors is a challenge due to the interactions of health care professionals’ behaviors with each other on the one hand and with ICT on the other. In addition, this model is dependent on human beings, cultural environments, and technological facilitators [50].

### Aim and Research Questions

The aims of this paper were to present the steps of the development of a KSMM for understanding how health care professionals share their knowledge; define the proper stages of achieving the best knowledge practices among health care professionals in medical imaging departments; and use the Delphi method to examine the reliability and validity among experts on that model [51].

Therefore, the key research questions were as follows: (1) what are the main components that structure the KSMM? (2) what are the stages of MM that managers in medical imaging departments need to follow to evaluate and enhance knowledge-sharing practices among health care professionals? (3) what are the indicators that control the KSMM, and how can managers measure them? and (4) how to evaluate the proposed KSSM regarding validity and reliability?

### Objectives

This paper had various objectives. The first objective is to identify the main stages and components that relate to the KSMM. The second objective is to explain the main characteristics of each stage involved in the MM in depth. The third objective is to create a proposal for an MM that includes the knowledge-sharing influencing indicators that affect knowledge-sharing practices among health care professionals in medical imaging departments, and to present the measurements for each indicator that form the components of the KSMM. Finally, the last objective is to generate consensus among the experts in knowledge management and senior managers of the medical imaging departments in the United Kingdom and Kuwait on the components of the MM along with its indicators and measurements to be implemented in all departments.

## Methods

### Overview

**Figure 1:**
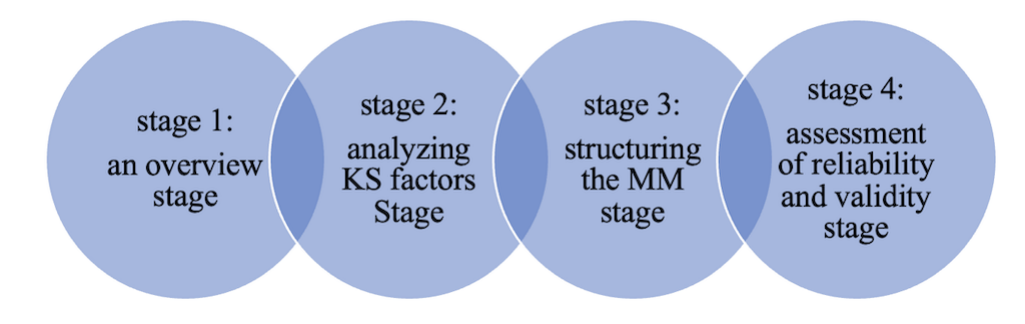
The 4 stages conducted in this study. KS: knowledge sharing; MM: maturity model.

This paper illustrates the development of an MM for knowledge sharing in medical imaging departments. There were four stages conducted in this study, as shown in Figure 1: (1) an *overview stage*, consisting of a review of previous literature that identified factors that affect knowledge-sharing practices in health care institutions in general and medical imaging departments specifically; (2) the *analysis of factors* stage, which consists of applying several methodologies to examine those factors; (3) the *structuring the MM* stage; and (4) the *assessment of reliability and validity* stage. Hence, the initial MM for knowledge sharing in the medical imaging department was developed.

### Ethical Considerations

This study followed the ethical guidelines outlined by the Christie hospital and the Kuwait Cancer Control Center (KCCC). Stage 1 was a systematic review, which did not require ethics approval. Stage 2 assessed potential factors with the involvement of health care professionals, and stage 3 analyzed the data from previous stages. The Christie hospital, following the University of Manchester regulations, did not require ethics approval for these two stages because no sensitive or personal question were asked; the KCCC, following regulation from the Ministry of Health, Kuwait, provided the approval (3797). Stage 4 used the Delphi method, which also does not require ethics approval because all information based on the respondents will be kept anonymous.

All respondents were asked to fill out the consent form electronically, as a mandatory request on the Qualtrics survey tool, before participating in the study. A brief description of the study was provided at the start of the electronic survey, followed by the consent form. Additionally, participants had a right to withdraw at any point of the survey based on the consent form provided. All the data are stored in the University of Manchester Qualtrics platform and kept anonymous, only accessible by the authors for the purposes of this study.

### Stage 1: An Overview

An overview stage illustrated the factors that affect knowledge sharing in medical imaging departments at the general hospital and cancer centers specifically. There were several studies that identified knowledge-sharing factors, which were divided into 3 categories: individual factors, administrative factors, and technological factors [42,52-64]. Any factors that have a positive impact on enhancing knowledge sharing are called facilitators. In contrast, any factors that hinder knowledge-sharing practices are called barriers.

### Stage 2: Analysis of Factors

In this stage, to examine and analyze those factors, mixed methods were used in this study. The mixed methods consist of questionnaires and semistructured interviews [65]. A concurrent, cross-sectional, triangulation, mixed methods design was used in this study in 2 cancer centers (the Christie hospital and KCCC). The questionnaire was structured based on the previous studies, and the semistructured interview questions were formed based on the factors in the previous stage to examine those factors by distributing questionnaires and conducting semistructured interview among health care professionals who are working in the medical imaging department at the 2 cancer centers [66-68]. We used self-selection sampling for the questionnaires and snowball sampling for the semistructured sampling techniques. The questionnaire was divided into 3 parts: the demographic section, knowledge-sharing practices, and factors that affect knowledge-sharing practices in the medical imaging departments. Multimedia Appendix 1 shows the survey questions. The questionnaires were distributed electronically using the Qualtrics (Qualtrics International Inc) survey tool. For the health care professionals working at the KCCC, a WhatsApp group (Meta Platforms) was used for distributing questionnaires, whereas the internal page was used as an official web-based tool for those questionnaires. The semistructured interviews were conducted via Microsoft Teams for the health care professionals in the medical imaging department of 2 cancer centers: the Christie hospital and KCCC.

The collection of the data was conducted between February 2023 and July 2023. After collecting the data, an analysis for the quantitative data were performed using Qualtrics Experience Management software package. The duration of the semistructured interviews was approximately 25 to 45 minutes. The thematic analysis was conducted to analyze the transcripts. All the codes and themes were analyzed using the NVivo software (Lumivero).

### Stage 3: Structuring the MM

In this stage, an MM for knowledge sharing was developed. On the basis of the identification and evaluation of factors that affect knowledge-sharing practices in medical imaging departments in previous rounds, a clear vision was developed to build a KSMM in a proper manner, helping the managers and policy makers to follow these steps and understand how these factors are related to each other in different stages. Furthermore, it helped them either to implement a knowledge-sharing environment or to identify the weak points of knowledge-sharing practices.

### Stage 4: Assessment of Reliability and Validity

In this stage, after the KSMM was developed, it was further assessed for reliability to validate the model. Without validation, the KSSM lacked reliability in its components. To apply this model, experts and senior managers should agree on its use. Therefore, this stage aimed to generate consensus among the experts in knowledge management and senior managers of the medical imaging departments in the United Kingdom and Kuwait on the components of the MM along with its indicators and measurements to be implemented in all departments. Consensus on the model contributes to updating and correcting missing information before reaching out to whom it concerns.

Our target was to recruit 7 to 10 participants (experts in knowledge management and senior managers in the medical imaging department). This is the minimum number of participants required to conduct this kind of study [69,70]. In addition, Cyphert and Gant [71] illustrated that the best results might be obtained by a homogenous group of 10 to 15 experts. There are no rigid rules in the Delphi method regarding selecting the size of the expert’s panel. However, the number of experts depends on the purpose of the study [72]. Delphi studies often use a sample size adjusted to the topic. By selecting experts with comparable broad understanding of the subject, a smaller sample size can be used [73]. We used the following four ways to choose participants: (1) we searched for the experts of knowledge management through the official university websites to find the senior lecturer in that field, especially at Kuwait University and the University of Manchester; (2) we searched through LinkedIn (Microsoft Inc) to find stakeholders for the National Health Service who have a role in knowledge management practices; (3) throughout the previous literature in knowledge management, there were several potential articles in this field with the authors name and contacts details, so we contacted them through this information to request their participation in this study; and (4) we asked the senior managers in the medical imaging department at the Christie hospital and KCCC to participate and help us to add their perspectives based on their professional experience. All participants remained anonymous during the research.

The modified Delphi method survey questions were generated based on the indicators and the measurements that structured the KSMM. The experts who participated in this study were informed of the aim, methods, and main components that structured the model to give them an overview of the study. The consensus survey questionnaire consisted the consent form (5 statements) and 3 sections: demographic questions (7 multiple-choice questions), a Likert scale with 5 points (disagree to agree) for the 17 indicators, and open-ended questions for it measurements to allow the experts to add their comments for updating the model. Study data were collected electronically using the Quartics survey tool and distributed via email. The use of email has a potential benefit in making it easier to maintain the anonymity of the respondents. Delphi studies ensure the anonymity of suggested comments and assessment to prevent group dynamics, strong personalities, or group conformism from influencing personal relationships between participants [74]. Descriptive statistics were used to analyze the Delphi studies, including SD, mean, and the intraclass correlation coefficient (ICC). We computed the mean, ICC, and SD based on the Likert scale of each indicator, where 1 indicates the least agreement and 5 indicates the highest agreement. In Delphi studies, IQRs are widely used to identify the level of group censuses [75,76]. However, we used mean, ICC, and SD in this study as they might be enough to identify the level of agreement for a model that was already structured.

## Results

### Stage 1: An Overview

On the basis of the previous literature, the factors that affect knowledge sharing in medical imaging departments are the same whether in a general hospital or a cancer center. However, they are different in terminology because the nature of the cancer center is mainly concerned with treating cancer cases, which require more than one specialty to end up with an appropriate protocol to treat specific cases [77]. Therefore, documenting the factors that affect knowledge sharing in medical imaging departments from the previous literature is important to test these factors in the next stage [77]. These factors are shown in Table 1 [77].

**Table 1:** Factors that affect knowledge sharing in the medical imaging departments in general hospitals versus cancer centers.

Types of factors	Facilitators terminologies in medical imaging departments in general hospitals	Facilitators terminologies in medical imaging departments in cancer centers
Individual factors	Trust Positive attitudes Awareness Experience Intrinsic motivation Personality Self-esteem Self-efficacy	Trust Positive attitudes Awareness Experience Intrinsic motivation Personality Self-esteem Self-efficacy
Departmental factors	CoP^a^ and interprofessional collaboration Leadership Culture Teamwork Extrinsic motivation Learning and training Physician rounds Departmental arrangements	MDT^b^ and community of oncologists Leadership Culture Teamwork Extrinsic motivation Learning and training Physician rounds Departmental arrangements
Technological factors	ICT^c^ (PACS^d^, social media, intranet, extranet, telemedicine, and teleradiology) Network Digital library	ICT (PACS, social media, intranet, extranet, telemedicine, and teleradiology) Network Digital library

^a^CoP: communities of practice.

^b^MDT: multidisciplinary team.

^c^ICT: information and communication technology.

^d^PACS: picture archiving and communication system.

### Stage 2: Analysis of Factors

A total of 85 responses were received (56 from the KCCC and 29 from the Christie hospital), and the response rate based on Quadrics was 100%. Qualtrics Experience Management software was used to analyze the quantitative data. The factors were divided into 3 categories based on the systematic review: individual factors, departmental factors, and technological factors [77]. The demographics characteristics of the 2 centers are shown in Table 2. The results of the questionnaires for the 2 cancer centers are provided in Multimedia Appendix 2.

The semistructured interviews were conducted online using Microsoft Teams with 13 health care professionals who were working in the medical imaging departments at the 2 cancer centers: 11 (85%) were from the KCCC and only 2 (15%) were from the Christie hospital. Thematic analysis was used to analyze the qualitative data. Codes were organized using NVivo software. This was done to validate the quantitative methods, understand their views, understand how knowledge-sharing practices were going on in these centers, and assess if there was a clear policy to adopt knowledge-sharing practices. Multimedia Appendix 3 shows the interview questions and consent form. All these methods were applied according to the ethical considerations at both canters.

**Table 2:** Demographic characteristics.

Characteristic			KCCC^a^ (n=56), n (%)		The Christie (n=29), n (%)
**Sex**					
	Male	26 (46)		7 (24)	
	Female	29 (52)		20 (69)	
	Prefer not to say	0 (0)		2 (7)	
**Age group (y)**					
	<20	0 (0)		1 (3)	
	20-30	3 (5)		8 (28)	
	30-40	19 (34)		7 (24)	
	40-50	25 (45)		8 (28)	
	50-60	7 (12)		3 (10)	
	>60	2 (4)		2 (7)	
**Educational level**					
	Diploma	7 (12)		4 (14)	
	First degree (Bachelor)	26 (47)		12 (41)	
	Master’s degree	11 (20)		8 (27)	
	Doctorate degree	9 (16)		0 (0)	
	Other	3 (5)		5 (17)	
**Work experience (y)**					
	<10	18 (32)		22 (76)	
	10-20	29 (51)		6 (21)	
	20-30	7 (12)		1 (3)	
	>30	2 (4)		0 (0)	

^a^KCCC: Kuwait Cancer Control Center.

The results revealed that there were 11 components derived from 5 categories, as shown in Table 3. These categories started with awareness, which is considered the main step to adopting knowledge-sharing practices in the department. Awareness of the importance of knowledge sharing in developing health care professionals’ skills and improving health care services is important to increase their willingness to share their knowledge. The results of the quantitative method showed that health care professionals in both cancer centers have a high level of awareness of the importance of knowledge sharing. The second category was related to the types of knowledge sharing. It is divided into 2 categories: tacit and explicit knowledge. Tacit knowledge is the most dominant type of knowledge in the medical imaging department because several meetings and specialized meetings (MDT meetings, community of practices, and community of oncologists) occur in these departments. In contrast, explicit knowledge exists in the endorsed documents, protocols, policies, and procedures manuals. Therefore, understanding both types of knowledge and organizing them is important to increase knowledge-sharing practices.

The third, fourth, and fifth categories were related to the factors that affect knowledge-sharing practices: individual, departmental, and technological factors. The mean scores of the factors fell between somewhat agree and strongly agree, which reveals that health care professionals believe in the importance of these factors in enhancing knowledge-sharing behaviors. The individual factors consisted of 2 components: communication among health professionals, and personality and positive attitudes. Building trusting relationships among health care professionals is important to allow them to share their experiences. In addition, most of the respondents agreed that intrinsic motivation has a positive impact on sharing knowledge by increasing their self-efficacy and self-esteem by giving them opportunities to practice their abilities and experiences. There is a strong relationship between personality, positive attitudes, and increased knowledge-sharing practices. The respondents indicated that knowledge-sharing behaviors occur among individuals. Each indivdual has a specific personality and attitude toward knowledge-sharing practices. Some of them like to share their knowledge in large groups, while others prefer when they share it in small groups.

**Table 3:** Categories, components, and indicators that build maturity model of knowledge sharing for the medical imaging departments.

Categories and components			Indicators
**Awareness**			
	Awareness and willingness	(1/17) Awareness and willingness toward KS^a^	
**Types of knowledge sharing: tacit knowledge and explicit knowledge**			
	Understanding and organizing a knowledge-sharing repository	(2/17) Structured and collected both types of knowledge	
**Individual factors:** **trust, positive attitudes, experience, intrinsic motivation, personality, self-esteem, and self-efficacy**			
	Communication among HCPs^b^	(3/17) Building trust among HCPs and sharing their experience (4/17) Increased intrinsic motivation (self-efficacy, and self-esteem)	
	Personality and positive attitudes	(5/17) Personality and communication among HCPs	
**Departmental factors: MDT^c^ and community of oncologists, leadership; culture, teamwork, extrinsic motivation, learning and training, physician rounds, and departmental arrangements**			
	Leadership and culture	(6/17) Structured leadership and creating culture (7/17) Handover policy	
	Achieving departmental tasks	(8/17) Creating teamwork	
	Continuous education and develop HCP skills	(9/17) Organizing (learning lectures, workshops, training sessions, physician rounds, and participation in conferences)	
	Decision-making	(10/17) Regular meeting (11/17) MDT and CoP^d^ decision-making	
	Infrastructure and workforce	(12/17) Meeting room and office layout (13/17) Enhanced extrinsic motivation (14/17) Organized work process	
**Technological factors: ICT^e^ (PACS^f^, social media, intranet, extranet, telemedicine, and teleradiology), network, and digital library**			
	Stored and shared patient data electronically	(15/17) Strong network (16/17) Implementation information, communication technology, and maintenance	
	Access to the electronical databases	(17/17) Digital library	

^a^KS: knowledge sharing.

^b^HCP: health care professional.

^c^MDT: multidisciplinary team.

^d^CoP: communities of practice.

^e^ICT: information and communication technology.

^f^PACS: picture archiving and communication system.

The departments have a big responsibility to enhance knowledge sharing based on respondents’ thoughts. Departmental factors consisted of 5 components: leadership and culture, achieving departmental task, continuous education, decision-making, and infrastructure and workforce. The participants agreed that a leader has a responsibility to create a culture of communication that allows health care professionals to practice knowledge-sharing behaviors. Developing health care professionals’ skills require setting a clear plan for practicing continuing education activities, such as attending conferences, lectures, training sessions, and workshops. Offering them space and time is vital for allowing them to practice knowledge-sharing activities by organizing tasks among them and giving them empty spaces. To achieve departmental tasks, working within a team is crucial to increase the number of tasks achieved. Decision-making regarding patient treatment is one of the important tasks in the department, which is usually undertaken in highly specialized meetings, such as MDTs and communities of practices.

Technological factors consisted of several technological modalities, such as PACS, social media, the intranet, the extranet, telemedicine, and teleradiology, that require high-speed networking. These factors consisted of 2 components: storing and sharing electronic data electronically, and access to electronic databases. The respondents illustrated that using technology requires skills for using it efficiently. Moreover, accessing the databases is vital to expanding health care professionals’ knowledge about up-to-date treatment plans that are used to treat patients with cancer.

### Stage 3: Structuring the MM

The development of the MM was derived from a systematic review that identified the factors affecting knowledge-sharing behaviors in the medical imaging department, as well as concurrent mixed methods that evaluated those factors in 2 cancer centers. The nonprobability sampling technique was used for mixed methods: the self-selection sampling technique for the quantitative method and the snowball sampling technique for the qualitative method. The results indicated that the KSMM consisted of 17 indicators divided into 11 components. These components represented the following 5 main categories: awareness, knowledge-sharing repository, individual factors, departmental factors, and technological factors. Table 3 shows the details of the MM indicators for knowledge sharing. Knowledge sharing is a dynamic process among health care professionals to manage the institutional process and interaction with ICT infrastructures. As a consequence, these components were incorporated into the 5 maturity levels. The levels were adopted from the work of Pee and Kankanhalli [19] that indicated that there are 5 levels of maturity in knowledge management (initial, aware, defined, managed, and optimized) based on people, process, and technology. The KSMM for the medical imaging department is presented in Multimedia Appendix 4. For each indicator, there are measurement questions that help managers and policy makers assess each indicator.

### Stage 4: Assessment of Reliability and Validity

A total of 9 different participants were recruited in this study: 9 in the first round and 9 in the second part. They were from 2 countries: the United Kingdom and Kuwait. Table 4 shows the demographic characteristics of the participants. Most of respondents were female (n=7, 78%), and they were mainly from Kuwait (n=7, 78%). Most (n=6, 67%) of the respondents had a doctorate degree and the rest (n=3, 34%) had first degree.

**Table 4:** Demographic characteristics of the participants in the Delphi method (N=9).

Demographic characteristics		Participants, n (%)
**Gender**		
	Female	7 (78)
	Male	2 (22)
**Age group (y)**		
	40-50	7 (78)
	50-60	2 (22)
**Highest education level**		
	First degree	3 (33)
	Doctorate degree	6 (67)
**Background**		
	Senior lecturer in knowledge managements	3 (33)
	Senior manager in medical imaging departments	6 (67)
**Experience (y)**		
	<10	1 (11)
	10-20	4 (44)
	20-30	4 (44)
**Country**		
	United Kingdom	2 (22)
	Kuwait	7 (78)

In the first round, most of the indicators and their measurements that formed the KSMM reached consensus with a narrow SD (<1); Multimedia Appendix 5 shows the level of consensus in both rounds. In contrast, indicators 1 (SD 1.25) and 17 (SD 1.15), which were about awareness and the digital libraries, respectively, did not reach to the consensus level with a large SD (>1). Therefore, the SD was more obvious and sensitive for this study to assess the level of consensus, compared to other measurements [74,78,79]. Awareness is the first component that forms the KSMM because it has a crucial role in enhancing knowledge-sharing practices among health care professionals. On the basis of their comments, there is a difference between the willingness to share knowledge and awareness. The willingness to share their knowledge comes from an awareness of the importance of knowledge sharing and believing in the value and benefits of their shared knowledge [80]. To increase awareness about knowledge sharing, the managers should set a clear policy to identify the benefits of sharing practices in their daily work to increase patient outcomes and reduce medical errors. In contrast, their willingness to share knowledge is directly related to their personality and how their intrinsic and extrinsic motivations encourage them to share their knowledge among other peers. On the basis of indicator 17, most (4/9, 44%) of the respondents indicated that the digital resources are more comprehensive among those indicators. The digital resources include digital databases that contain libraries that play an important role in enhancing their knowledge in any field and therefore increasing knowledge-sharing practices. Regarding other measurements, they showed less sensitivity compared to the SD because they did not indicate any disagreements compared to the SD. Therefore, the SD is a more sensitive measurement compared to others [79].

Besides the analysis of the Likert scale, we received an important comment that helped to update the KSMM based on their experiences. Multimedia Appendix 6 shows the comments of the participants and our response to update the model.

In round 2, all the components of KSMM met the consensus SD level (<1.0). This indicates that all the respondents agreed with the updated version of the model that kept all their comments and considerations. Therefore, there was no follow-up round for this study because consensus on all the indicators along with their measurements of the KSMM was met. The final KSMM in the medical imaging department is presented in Multimedia Appendix 4.

## Discussion

### Principal Findings

Despite the considerable attention given to knowledge management in health care institutions and the significance of the MM in managing health care resources, knowledge sharing in hospitals has been less focused on developing the MM as a tool for assessing knowledge-sharing practices or a road map to adopting knowledge-sharing behaviors. This is the essence of this work. This study aimed to develop a KSMM in the medical imaging department by assessing the factors that affect knowledge-sharing practices. Several MMs have been developed for social media, health systems, digital libraries, ICT, PACS, and telemedicine [43,45,47-49]. These models were used to assess each factor that affects knowledge-sharing practices in benchmarking efforts and to develop progressive strategies that might improve its activities. In addition, Liu et al [46] shed light on a MM for multidisciplinary cancer teams. Their model consisted of 17 indicators that were used to measure health care professionals’ performance and monitor the quality of performance at cancers centers over time.

**Figure 2:**
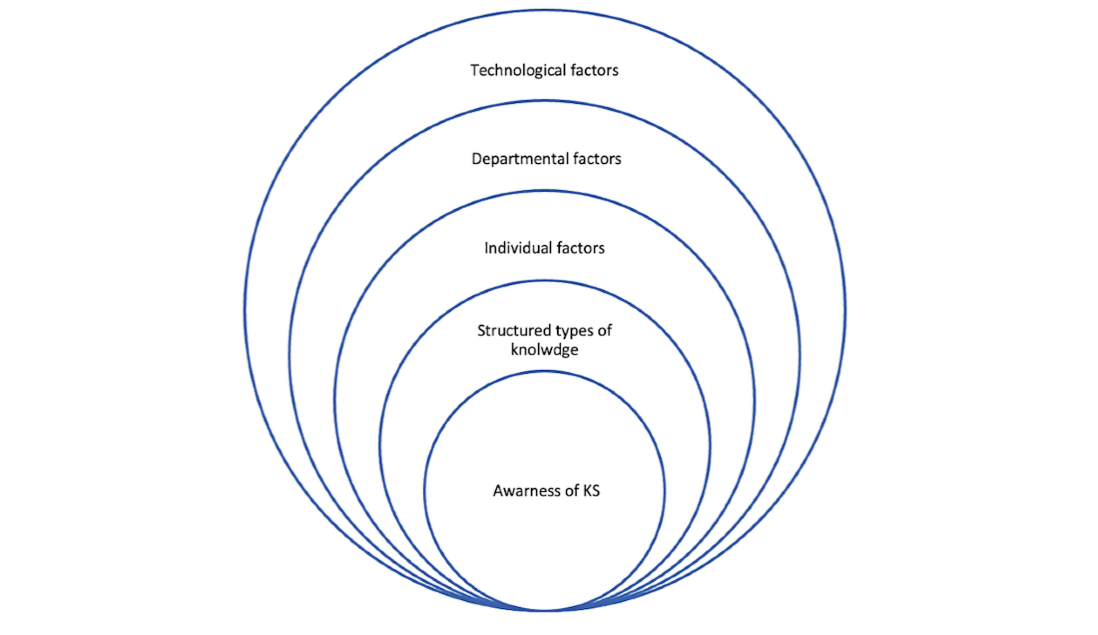
Schematic description of the 5 components that affect the knowledge sharing maturity model in medical imaging departments. KS: knowledge sharing.

Figure 2 highlights the 5 components that affect the KSMM in the medical imaging department and classifies each component in terms of its influence on knowledge-sharing behaviors. Awareness of the importance of knowledge management is one of the core components that contribute to the adoption of knowledge-sharing practices among health care professionals. Most of the respondents showed a high level of awareness of the importance of knowledge sharing in developing their skills, increasing health care services, and reducing medical errors. Therefore, results showed that more than half of respondents in both cancer centers (the Christie: 17/29, 59%; KCCC: 32/56, 57%) participated daily in knowledge-sharing activities that were available in their department. In addition, one of the articles showed that without awareness of the importance of knowledge sharing, there were no knowledge-sharing practices in the medical imaging department [13]. The next step was structuring the types of knowledge (tacit and explicit). Understanding the types of knowledge available in the department and how to capture, document, and share it is vital to accelerating knowledge-sharing practices in the department. Tacit knowledge appears in the medical imaging department as a dominant type among health care professionals that is considered a tool for sharing knowledge in lectures, conferencing, and meetings. In contrast, explicit knowledge takes several forms, such as documents, policies, procedures, and manuals. This form allows workers to reach it anytime in an easy way [81]. From the third to the fifth step, factors that affected knowledge sharing were divided into 3 categories: individual factors, followed by departmental factors, and finally technological factors [13,42,52-55,57,59-62].

The structure of the KSMM relies on the deep interpretation of several methods. The KSMM was built based on the interpretation of the systematic review and the concurrent mixed methods. That model is lacking in validation. Applying the modified Delphi method helps to validate this model by involving experts. In the first round, the results revealed that indicators 1 and 17 had a large SD (>1).

The first round provided several comments and suggestions to modify the model. There were significant comments that needed to be considered and updated before being shared with the participants in the second round. Some of the respondents (R6 and R7) indicated that tacit knowledge is different from explicit knowledge in optimization, so the updated model indicated the way to optimize tacit knowledge in a significant way. Tacit knowledge is intangible, which is difficult to codify and capture [82]. To optimize and capture tacit knowledge, it needs to be part of an externalization process that focuses on transforming the tacit knowledge into explicit knowledge [83]. There are several methods of externalization, for example, recording the meeting and writing a summary note to make it easy to share it with health care professionals. On the basis of indicator 3, the respondents (R1, R6, and R7) illustrated that there are 2 types of trust that we should be aware of. Trust in their abilities and other expertise helps them perform more tasks in the working area, and benevolent trust helps to reduce conflicts among them and, therefore, increases knowledge sharing. The trust itself among group members might be increased throughout social activities more than task communication [84]. In addition, Chen and Hung [85] illustrated that both types of trust help increase the desire to share their knowledge, resulting in enhancing the level of positive outcomes in shared groups. Therefore, leaders and managers should be aware of social activities that help increase their trust, which is defined as a level of confidence among members. Regarding individual facilitators, a respondent (R7) illustrated that there is a difference between self-efficacy and self-esteem. Chan and Hung [85] have defined knowledge-sharing self-efficacy as an individual’s confidence in their knowledge, abilities, and experience to achieve several tasks that might be helpful for other peers. A high level of self-efficacy is directly related to a higher level of performance on tasks [85]. In contrast, self-esteem is defined to play a key role in emotional and behavioral adjustment, as well as academic achievements [86]. Leaders and senior managers should encourage their employees to share their knowledge, helping them to understand the importance of sharing knowledge among them and how it has direct positive outcomes—by understanding their abilities and increasing their awareness of knowledge sharing, and not by forcing them. Giving them positive feedback on their abilities directly increases their self-efficacy and self-esteem [80,87].

Regarding applying knowledge-sharing practices, the respondents (R1, R5, R6, and R7) indicated that the senior managers and leaders should be aware of the knowledge-sharing models. There are several models for knowledge sharing, and each of them has a certain characteristic [36,88-90]. In addition, they must be able to create a knowledge-sharing model based on the resources that are available in the department. On the basis of the indicator 7, a respondent (R5) indicated that the handover policy should be applied and used not only when health care professionals decide to leave the department. They must share their knowledge with other peers in routine work to allow them to cover their work when they are absent. Such behavior helps to make knowledge-sharing practices more active in daily work. On the basis of teamwork, a respondent (R7) illustrated that to maximize the level of knowledge sharing, leaders and managers should be aware of the diversity of the teamwork in the workplace and the diversity of the member professionals in one teamwork [91]. The diversity is directly related to the individual differences in their knowledge and the abilities that make them unique from other peers [91]. The diversity of the member professionals in one teamwork helps to increase their performance by bringing new knowledge and experiences. However, diversity in one teamwork has several challenges, such as increasing conflicts and a lack of trust. In contrast, the diversity of teamwork who are performing the task has a positive impact on sharing knowledge.

On the basis of indicator 10, which illustrates meetings, the respondents (R1, R5, R6, and R7) indicated that there are formal and informal meetings in the department, and informal meetings have a significant role in increasing knowledge-sharing behaviors. Hutchinson and Qunitas [92] have defined informal meetings as meetings that indicate knowledge-sharing practices without focusing on the task and the target that is directed by the organization. However, the leader should be aware of the difference between formal and informal meetings and strike a balance between them to enhance knowledge-sharing practices.

Indicator 15 illustrated the strong network. A strong network plays a vital role in enhancing knowledge sharing by introducing new technology to store and share data, anytime and anywhere. A respondent (R7) identified that artificial intelligence (AI) is an important technology, playing an important role in increasing efficiency, effectiveness, and productivity, and therefore increasing knowledge sharing [93]. It is defined as a set of ICTs that mimic human intellect with the goal of enhancing jobs, increasing efficiency, and boosting economic growth [94]. In addition, Arakpogun et al [94] indicated that the benefit of using AI is limited, because new knowledge needs to be introduced as part of the AI learning process, and therefore, more effective collaboration with knowledge-sharing systems is needed.

The Delphi method has limitations, including the use of nonrandomized samples; subjectivity and bias from expert panel composition; and a lack of consensus recommendations for the number of participants, rounds, and reporting criteria [69]. In this study, a significant limitation was the small size of the panel in 2 rounds and only in 2 countries. Despite these limitations, the modified Delphi method is an effective tool for validating several models.

Through this study, the key research questions were answered. The model can be used as a scoring tool to assess knowledge-sharing practices, with each MM scoring 1, 2, 3, 4, and 5, respectively. Moreover, this model can help managers and policy makers find opportunities for improvements and ways to achieve them. In addition, applying the KSMM helps health care institutions increase health services and patient outcomes, reduce errors, and solve the problem in a practical way. The Delphi study identified several comments and suggestions that helped in improving the KSMM. All these comments were kept in consideration for updating the model because they were based on participants’ experiences in the workplace as either a senior manager in knowledge managements or a lecturer in the knowledge management field. Multimedia Appendix 4 shows the new KSMM with its updates.

### Conclusions

Knowledge sharing is considered a core step in the implementation of knowledge management. Health care institutions have a responsibility to adopt knowledge-sharing practices to manage knowledge that is either tacit or explicit. A medical imaging department is crucial to any health care institution. Therefore, creating the KSMM is important to develop knowledge-sharing practices. The model proposed in this study allows managers to measure the maturity level of knowledge sharing in the medical imaging department. By providing the road map, the KSSM allows policy makers and managers in health care to appraise knowledge-sharing practices and adopt a culture of knowledge sharing to achieve departmental tasks and improvements. In addition, it could help managers assess knowledge-sharing practices in the medical imaging department and find the weak points that have a negative impact on those behaviors. A range of factors were addressed in our previous work, and we then evaluated those factors in 2 medical imaging departments in 2 cancer centers. The factors were further divided into 5 categories. Therefore, the KSMM consisted of 17 indicators that were divided into 11 components and presented in 5 categories. The most important of these indicators is awareness of the importance of knowledge sharing, presumably because it allows health care professionals to develop their skills and perform several tasks. The KSMM went through 2 rounds to ensure consensus among the experts who participated in the Delphi method. Regarding the measurements, all the indicators reached the consensus level with SD <1. Therefore, the model is ready to be applied in the medical imaging departments, and with minor modifications based on their resources, it might be applied in any institution. The model presented might also be used as a reference for improving knowledge-sharing practices. The measurement of each indicator helps the managers assess what level they are at. If the answer to the first level is no, then they must work on their current level until they achieve it and then move on to the next level. It enables them to share their experience in knowledge-sharing practices and serve as a lesson learned for another department with lower KSMM results. In the future, we are looking forward to comparing knowledge-sharing practices before and after implementing the KSMM in the medical imaging department. In general, knowledge-sharing practices are important in health care institutions to avoid repetitive errors, improve health care services, improve collaboration and communication among staff, and therefore encourage them to come up with new ideas together.

## Appendix

Multimedia Appendix 1 The survey questions and consent form.

Multimedia Appendix 2 Results of the factors that affect knowledge sharing in both cancer centers.

Multimedia Appendix 3 The interview questions and consent form.

Multimedia Appendix 4 The details of the maturity model for knowledge sharing in medical imaging departments.

Multimedia Appendix 5 The level of consensus in both rounds.

Multimedia Appendix 6 The respondents’ comments with our response.

## Abbreviations

AI: artificial intelligence
ICT: information and communication technology
KCCC: Kuwait Cancer Control Center
KSMM: knowledge sharing maturity model
MDT: multidisciplinary team
MM: maturity model
PACS: picture archiving and communication system
