# Supplementary material for: Knowledge Sharing Maturity Model for Medical Imaging Departments: Development Study
Source: JMIR Hum Factors. 2025 May 6;12:e54484. doi: 10.2196/54484 (PMC12093077; doi:10.2196/54484)
Supplement: Multimedia Appendix 2 [file humanfactors_v12i1e54484_app2.docx]

| **Multimedia appendix 2: Results of the factors that affect knowledge sharing in both cancer centers.** | | | | | | |
| --- | --- | --- | --- | --- | --- | --- |
| Factors | | | KCCC^a^ score, mean | Level of agreement | The Christie score, mean | Level of agreement |
| **Individual factors** | | | | | | |
|  | At the hospital, there are periodic meetings in which employees working in different disciplines may participate. | | 5.64 | Agree | 5.17 | Somewhat agree |
|  | At the hospital, there are continuous education programs, such as training courses and workshops within the hospital, in which employees can participate. | | 5.87 | Agree | 5.44 | Agree |
|  | Total | | 5.7 | Agree | 5.51 | Agree |
|  | **Trust** | | | | | |
|  |  | I feel fully confident in my own knowledge, and I want to share it with others. | 5.96 | Agree | 5.6 | Agree |
|  |  | I trust in the knowledge of my colleagues and the information that they have shared with me. | 5.82 | Agree | 5.72 | Agree |
|  |  | If I share my knowledge, my colleagues will feel confident about my ideas, skills, and capabilities to enhance knowledge sharing. | 5.76 | Agree | 5.6 | Agree |
|  |  | If I face problems during work, my colleagues will try to help me solve them. | 6.3 | Strongly agree | 5.2 | Somewhat agree |
|  |  | Total | 6 | Agree | 5.81 | Agree |
|  | **Awareness** | | | | | |
|  |  | I am aware of the importance of knowledge sharing among employees in the workplace. | 5.17 | Somewhat agree | 6.10 | Agree |
|  |  | Knowledge sharing among employees helps prevent mistakes that could happen during day-to-day work. | 6.35 | Strongly agree | 6.17 | Agree |
|  |  | Sharing knowledge with my colleagues will help me gain new skills and ideas. | 6.44 | Strongly agree | 6.10 | Agree |
|  |  | Knowledge-sharing behaviors help employees learn faster. | 6.42 | Strongly agree | 6.13 | Agree |
|  |  | Total | 6.42 | Strongly agree | 5.48 | Agree |
|  | **Positive attitudes** | | | | | |
|  |  | I believe that positive attitudes will help enhance knowledge sharing. | 5 | Somewhat agree | 6.51 | Strongly agree |
|  |  | Positive attitudes play a significant role in increasing knowledge-sharing behaviors. | 6.5 | Strongly agree | 6.13 | Agree |
|  |  | I believe that positive attitudes are the first step to sharing knowledge. | 6.4 | Strongly agree | 5.65 | Agree |
|  |  | Total | 6.4 | Strongly agree | 6.16 | Strongly agree |
|  | **Experience** | | | | | |
|  |  | I have a good amount of experience that I can share with my colleagues. | 6.14 | Strongly agree | 6.55 | Strongly agree |
|  |  | I believe that experience plays a significant role in sharing knowledge. | 6.33 | Strongly agree | 5.89 | Agree |
|  |  | Knowledge-sharing behaviors will increase when employees have enough experience. | 6.28 | Strongly agree | 5.40 | Agree |
|  |  | Total | 6.2 | Strongly agree | 5.77 | Agree |
|  | **Personality** | | | | | |
|  |  | I have confidence in my ability to share knowledge. | 6.07 | Agree | 6.03 | Agree |
|  |  | I enjoy sharing my knowledge with colleagues. | 6.12 | Agree | 6.17 | Strongly agree |
|  |  | I am open-minded and receptive to new ideas. | 6.26 | Strongly agree | 6.34 | Strongly agree |
|  |  | Total | 6.15 | Agree | 6.18 | Strongly agree |
|  | **Self-esteem** | | | | | |
|  |  | I believe that self-esteem is an important aspect in sharing knowledge. | 6.3 | Strongly agree | 5.96 | Agree |
|  |  | I have confidence in my ability to successfully share knowledge with colleagues. | 6.19 | Strongly agree | 6.03 | Agree |
|  |  | Total | 6.25 | Strongly agree | 4.75 | Somewhat agree |
|  |  | **Self-efficacy** | | | | |
|  |  | I believe that self-efficacy is important to motivate us to share knowledge. | 6.19 | Strongly agree | 5.75 | Agree |
|  |  | I have the self-efficacy to share my knowledge with others. | 6.10 | Agree | 6.68 | Strongly agree |
|  |  | Total | 6.15 | Agree | 5.92 | Agree |
|  | **Intrinsic motivation** | | | | | |
|  |  | I believe that I have knowledge that will help in increasing productivity. | 5.9 | Agree | 6.17 | Strongly agree |
|  |  | I feel happy when I am helping my colleagues by sharing my knowledge with them. | 6.10 | Agree | 6.10 | Agree |
|  |  | Total | 5.96 | Agree | 5.89 | Agree |
| **Departmental factors** | | | | | | |
|  | **Extrinsic motivation** | | | | | |
|  |  | There is acknowledgment for employees who share their knowledge from the hospital. | 5.3 | Agree | 4.06 | Neither agree nor disagree |
|  |  | Sharing knowledge will help me advance in my career. | 5.96 | Agree | 4.68 | Somewhat agree |
|  |  | Total | 5.66 | Agree | 4.37 | Neither agree nor disagree |
|  | **Leadership** | | | | | |
|  |  | I believe that the hospital leadership has a responsibility to encourage and improve knowledge-sharing activities. | 6.23 | Strongly agree | 6.48 | Strongly agree |
|  |  | I believe that the leadership plays an important role in minimizing conflict. | 6.28 | Strongly agree | 6.37 | Strongly agree |
|  |  | The head of department or senior management has a positive impact on enhancing knowledge sharing. | 6.23 | Strongly agree | 5.75 | Agree |
|  |  | Total | 6.25 | Strongly agree | 6.20 | Strongly agree |
|  | **Teamwork** | | | | | |
|  |  | I believe that teamwork plays a significant role in sharing knowledge. | 6.39 | Strongly agree | 6.48 | Strongly agree |
|  |  | Teamwork is a part of daily work in each department that enhances knowledge sharing. | 6.44 | Strongly agree | 6.24 | Strongly agree |
|  |  | Teamwork has a positive impact on enhancing well-being among employees. | 6.41 | Strongly agree | 6.44 | Strongly agree |
|  |  | Total | 6.41 | Strongly agree | 6.39 | Strongly agree |
|  | **Culture** | | | | | |
|  |  | I believe that a culture of communication is important to enhance knowledge sharing. | 6.21 | Strongly agree | 6.41 | Strongly agree |
|  |  | Cultural collaboration plays a significant role in sharing knowledge among employees. | 6.07 | Agree | 6.17 | Strongly agree |
|  |  | Total | 6.18 | Strongly agree | 6.29 | Strongly agree |
|  | **Community of practice** | | | | | |
|  |  | There are communities of practice at the hospital that I can you for knowledge sharing. | 5.41 | Agree | 4.93 | Somewhat agree |
|  |  | I believe that communities of practice play a significant role in enhancing knowledge sharing among employees. | 5.64 | Agree | 4.34 | Neither agree nor disagree |
|  |  | Multidisciplinary team meetings are important to increase patient outcomes and reduce errors. | 6.1 | Agree | 6.10 | Agree |
|  |  | Total | 5.66 | Agree | 5.42 | Agree |
|  | **Learning and training** | | | | | |
|  |  | I believe that workshops have a significant impact on knowledge sharing. | 6.12 | Agree | 5.65 | Agree |
|  |  | At the hospital, there are workshops and training sessions that enhance my learning and knowledge sharing. | 5.92 | Agree | 5.17 | Somewhat agree |
|  |  | The hospital encourages employees to participate in conferences locally and internationally. | 5.42 | Agree | 4.62 | Somewhat agree |
|  |  | I believe that morning meeting sessions have a positive impact on knowledge sharing. | 5.98 | Agree | 5.58 | Agree |
|  |  | Total | 5.79 | Agree | 5.25 | Somewhat agree |
|  | **Departmental arrangements** | | | | | |
|  |  | At the hospital, there is a conference room or meeting room that can be used for knowledge sharing. | 6.07 | Agree | 5.48 | Agree |
|  |  | I believe that offering an open space to share knowledge is part of a department’s responsibility. | 6.12 | Agree | 5.37 | Somewhat agree |
|  |  | At the hospital, knowledge-sharing practices are part of daily working practice. | 6.07 | Agree | 5.03 | Somewhat agree |
|  |  | Total | 6.1 | Agree | 5.29 | Somewhat agree |
|  | **Physician rounds** | | | | | |
|  |  | I believe that daily physician rounds are an important way of improving knowledge. | 5.89 | Agree | 5.31 | Agree |
|  |  | At the hospital, there are daily rounds for professional employees to help develop skills. | 5.16 | Somewhat agree | 4.68 | Somewhat agree |
|  |  | Total | 5.64 | Agree | 4.82 | Somewhat agree |
| **Technological factors** | | | | | | |
|  | **ICT^b^** | | | | | |
|  |  | There is ICT infrastructure (eg, intranet, extranet, and PACS^c^) at the hospital. | 5.8 | Agree | 6.34 | Strongly agree |
|  |  | I believe that social media has a significant impact on knowledge-sharing behaviors. | 5.58 | Agree | 4.51 | Somewhat agree |
|  |  | There are technical support and maintenance groups available to address ICT-related problems. | 6.62 | Strongly agree | 5.58 | Agree |
|  |  | Employees at the hospital have the knowledge and skills to use ICT effectively. | 5.62 | Agree | 5.58 | Agree |
|  |  | Employees at the hospital use social ICT to communicate with each other. | 5.57 | Agree | 4.96 | Somewhat agree |
|  |  | Total | 5.62 | Agree | 5.20 | Somewhat agree |
|  | **Network** | | | | | |
|  |  | At the hospital, there is a high-speed network available. | 5.62 | Agree | 5.20 | Somewhat agree |
|  |  | I believe that an available network is vital in enabling knowledge sharing. | 6.64 | Strongly agree | 5.82 | Agree |
|  |  | Total | 5.33 | Agree | 5.56 | Agree |
|  | **Digital libraries** | | | | | |
|  |  | I believe that digital libraries facilitate learning and, therefore, knowledge sharing. | 5.91 | Agree | 5.62 | Agree |
