# Supplementary material for: Knowledge Sharing Maturity Model for Medical Imaging Departments: Development Study
Source: JMIR Hum Factors. 2025 May 6;12:e54484. doi: 10.2196/54484 (PMC12093077; doi:10.2196/54484)
Supplement: Multimedia Appendix 4 [file humanfactors_v12i1e54484_app4.docx]

| Categories, components, and indicators | | | Level 1: initial | | | Level 2: aware | | | Level 3: define | | | Level 4:  Managed | | | Level 5: optimized | | |
| --- | --- | --- | --- | --- | --- | --- | --- | --- | --- | --- | --- | --- | --- | --- | --- | --- | --- |
| Awareness^a^ | | | | | | | | | | | | | | | | | |
|  | Awareness toward KS^b,c^ | | | The term knowledge sharing is not new. HCPs^d^ are not aware of or understand the importance of knowledge sharing. | | | HCPs are aware of the importance of knowledge sharing in enhancing their skills and increasing patient outcomes, but others have miss understood. | | | HCPs are aware of the importance of knowledge sharing, and the facilitators and barriers that affect knowledge-sharing practices. | | | Circular discussions and meetings are organized to introduce the concept of knowledge sharing. | | | HCPs are fully aware of the importance of knowledge sharing, and the factors that affect it. They are willing to share their knowledge with others. | |
|  | Measurements | | | Do HCPs know about knowledge sharing in general?  Are they willing to understand the concept of knowledge sharing? | | | Has level 1 achieved?  Are HCPs aware of the importance of knowledge sharing? | | | Has levels 1 and 2 achieved?  Do you know the definition of knowledge sharing?  Do HCPs knew about facilitators and barriers that affect knowledge-sharing practices? | | | Has levels 1, 2, and 3 achieved?  Are there any plans to introduce and discuss the importance of knowledge sharing? | | | Has level 1, 2, 3, and 4 achieved?  Is there a clear policy that shows the importance of understanding knowledge-sharing practices at the workplace? | |
| **Types of knowledge sharing^e^** | | | | | | | | | | | | | | | | | |
|  | Understanding and organizing knowledge-sharing repository | | | | | | | | | | | | | | | | |
|  |  | Structured, and collected both types of knowledge^f^ | | | HCPs and their managers are not aware of 2 types of knowledge (tacit and explicit). In addition, they do not know what kind of knowledge they have. | | | HCPs are aware of the tacit knowledge that they have, such as thoughts, skills and ideas based on their working experience. In addition, they are aware of the explicit knowledge that it exits in the medical imaging department, such as policies, manuals, procedures, patient reports, quality control documents, and incident report. However, it is not organized. | | | HCPs are aware of both types, and how to share, and discriminate between them to create new knowledge. | | | Senior managers and heads of departments are provided a place and offered the time to share their thoughts and ideas, which is tacit knowledge. For example, circular discussion in a, morning or daily or weekly or monthly meeting.  Explicit knowledge is organized based on its purpose. For example:  Policies: department guidelines, ethical process, incident reports, hand over policies, and quality control.  Patients data: patients’ data, scans, history, and results. | | | HCPs now fully understand both types of knowledge, what are the purpose of them, and how to use and access them to enhance knowledge-sharing practices.  Managers have the ability to optimize the explicit knowledge by setting clear policies for any indicators and tasks in the departments by documenting them. In addition, they have an ability to optimize the tacit knowledge by the externalization process that focused on converting the meeting’s talk to the documents notes for easy sharing among them and for those who missed those meetings. |
|  |  | Measurement | | | Do HCPs and their managers know about types of knowledge? | | | Has level 1 achieved?  Are HCPs aware of the types of knowledge either tacit or explicit that exist in the department? | | | Has levels 1 and 2 achieved?  Are HCPs aware of both types of knowledge and able to discriminate between them? | | | Has levels 1, 2, and 3 achieved?  Is there time and regular meetings to share (tacit and explicit) knowledge?  Are there clear classifications for the various types of explicit knowledge that exits the department? | | | Has levels 1, 2, 3, and 4 achieved?  Is there a clear policy for explaining both types of knowledge to support knowledge-sharing behaviors among HCPs?  Are the explicit and tacit knowledge optimized by the managers in the department? |
| **Individual factors^g^** | | | | | | | | | | | | | | | | | |
|  | Communication among HCPs | | | | | | | | | | | | | | | | |
|  |  | Building trust among HCPs, and sharing their experience^h^ | | | There is no trust among HCPs or their managers, and they are not aware of the importance of trust in sharing knowledge.  There is no trust on their competencies, and colleagues’ expertise.  There are several problems and conflicts among each other in daily work. | | | HCPs are aware of the importance of trust in their knowledge sharing in enhancing their experiences, and importance of the benevolence trust in minimizing their conflicts. However, there is a lack of trust in their abilities, and experiences to share their knowledge, and they doubt their abilities. | | | HCPs trust their knowledge, experience, and skills as well as those of others. | | | Skills and experiences are shared by HCPs because they trust each other to increase patient outcomes and reduce errors. | | | Managers and HCPs fully understand the importance of trust, regarding other experiences that they have shared with others. In addition, managers trust their HCPs to perform procedures efficiently. |
|  |  | Measurements | | | Is there benevolent trust among HCPs or between their managers?  Is there trust on their ability to perform the departmental tasks?  Are there conflicts among HCPs in the department? | | | Has level 1 achieved?  Are HCPs aware of the importance of trust in their competencies, and other expertise in enhancing the sharing of their experiences?  Are HCPs aware of the importance of trust benevolence in minimizing the conflicts among them. | | | Has levels 1 and 2 achieved?  Are HCPs fully trusting their knowledge, peer’s knowledge, and shared experiences? | | | Has levels 1, 2, and 3 achieved?  Are HCPs sharing their knowledge and experiences in their daily work to improve health services? | | | Has levels 1, 2, 3, and 4 achieved?  Are managers trusting HCPs skills and experiences?  Is the department free from any conflicts? |
|  |  | Increased intrinsic motivation (self-efficacy and self-esteem)^i^ | | | There is no inner feeling, self-efficacy, and self-esteem that help HCPs to seek out optimal challenges and share their knowledge based on their abilities and interests. | | | There is intrinsic motivation, self-efficacy, and self-esteem, and managers are aware of the importance of those in enhancing knowledge sharing, but there is lack of opportunities that help them to use their skills.  HCPs are aware of the importance of self-efficacy, which is related to their feelings toward their feelings in performing the departmental task successfully.  HCPs are aware of the importance of self-esteem in building the positive evaluation on their selves; therefore, that pushes them to achieve more tasks. | | | HCPs have an internal factor (joy and satisfaction) that cause knowledge-sharing behaviors. Each HCP has high self-esteem and self-efficacy that allows them to believe in their self and to be capable to achieve institutional tasks, and procedures. | | | Clear policy is created to help them to increase their motivation, self-efficacy, and self-esteem, for example: giving them opportunities to use their skills by allowing them to set new procedures and protocols that contribute to increasing health services, giving them positive feedback that help them to seek optimal achievements in their tasks.  There are equal opportunities that help them to use their abilities to achieve the tasks.  There are several self-appreciations that help them to increase their self-esteem, such as (giving them thankful certificate, and putting their name on their work). | | | Managers and HCPs fully understand the importance of self-efficacy and self-esteem to accelerate knowledge-sharing practices among them. In addition, HCPs who have high self-efficacy and self-esteem are more willing to share their knowledge compared with others.  Managers have a clear positive self-talk, well planned goal-settings, and constructive feedback that help HCPs to increase their confidents, intrinsic motivation, and therefore increase knowledge-sharing practices. |
|  |  | Measurement | | | Do HCPs have a good self-efficacy that help them to perform the tasks successfully?  Do HCPs have a good level of self-esteem that help them to seek out optimal challenges? | | | Has level 1 achieved?  Are managers aware of the importance of self-efficacy, and self-esteem in enhancing knowledge sharing?  Are there opportunities that allow them to increase their self-efficacy that help them to use their skills successfully?  Do HCPs have positive evaluation on their ability based other feedback that help them to increase their self-esteem, and therefore increase knowledge-sharing practices. | | | Has levels 1, and 2 achieved?  Are HCPs capable to achieve departmental tasks and procedures? | | | Has levels 1, 2, and 3 achieved?  Are their clear policies to enhance their abilities?  Are their equal opportunities for HCPs that help them to boost their efficacy to achieve the goals?  Does their self- appreciations help them to increase their self-esteem? | | | Has levels 1, 2, 3 and 4 achieved?  Are managers fully able to understand the importance of self-efficacy and self-esteem to accelerate knowledge-sharing practices among them, and do they have a clear plan for it? |
|  | **Personality and positive attitudes** | | | | | | | | | | | | | | | | |
|  |  | Personality and communication among HCPs^j^ | | | The managers are not aware of the importance of personality in enhancing knowledge sharing. In addition, there are no positive attitudes among HCPs and their managers. | | | The managers are aware of the types of personality that are divided into 5 types: extraversion, eroticism, openness to experience, and moods and emotions. However, they are not aware of the relationship between personality and positive attitudes toward enhancing knowledge sharing practices. | | | Each HCP has a specific personality that cannot be changed in the workplace because it was formulated where the person was raised. In addition, the managers are aware of the importance of personality and positive attitudes in enhancing knowledge sharing. | | | HCPs at the workplace respect each other no matter what kind of personality the person has and treat each other with a positive attitude. In addition, managers give them the chance to develop their personalities regarding communicating with each other to enhance knowledge-sharing practices and solve their problems. | | | Managers and health care workers fully understand the importance of positive attitudes in enhancing knowledge sharing by allowing them to share their experience, thoughts, and ideas with total respect to their personalities because the main target in the workplace is to increase patient outcomes and reduce errors. |
|  |  | Measurement | | | Do HCPs have positive attitudes, and good personality among their peers? | | | Has level 1 achieved?  Are managers aware of the types of personalities that directly affect communication among them? | | | Has levels 1, and 2 achieved?  Are managers aware of the importance of positive attitudes in sharing knowledge?  applying Com\b model to change the behaviors to positive attitudes, are managers aware of Com/B model to change their behaviors to positive attitudes? | | | Has levels 1, 2, and 3 achieved?  Are managers giving HCPs chances to develop their personalities?  Are HCPs respectful to each other in the workplace? | | | Has 1evels 1, 2, 3, and 4 achieved?  Is there any conflict among workers and managers?  Are there any activities that allow them to develop their personalities? |
| **Departmental factors^k^** | | | | | | | | | | | | | | | | | |
|  | Leadership and culture | | | | | | | | | | | | | | | | |
|  |  | Structured leadership and creating culture^l^ | | | The managers or senior departments do not have enough knowledge about modeling knowledge sharing to create a culture for communication in their department. | | | There is a manager and a head of department, but they are not aware of the role of leadership in increasing knowledge-sharing practices. | | | All health care works are aware of the role of leadership in facilitating individual and collective efforts to understand and influence the people to realize what is to be done and how to realize shared objectives. | | | Leaders create a culture that consists of the values and beliefs that directly affect the institutional tasks and procedures. In addition, they set several policies that organize the workflow flow, solving their problems to optimize knowledge-sharing practices within the departments. | | | Leaders fully understand their role in creating a culture of knowledge sharing by understanding the variety of models in knowledge sharing and how to adopt them or create new one based on their resources. After that, provide them a space to share their knowledge. |
|  |  | Measurement | | | Is there a leader in the department?  Are they aware of types of models of knowledge-sharing behaviors, and how to create one based on resources in their department? | | | Has level 1 achieved?  Are leaders aware of their role in enhancing knowledge-sharing practices? | | | Has levels 1, and 2 achieved?  Are HCPs aware of their leadership role in facilitating resources to help HCPs reach their objectives? | | | Has levels 1, 2, and 3 achieved?  Are the leaders having clear policies to organize knowledge-sharing practices? | | | Has levels 1, 2, 3, and 4 achieved?  Is there a specific model of knowledge sharing that they follow?  Are the tasks done based on the workflow plan?  Is the leader fair in giving the worker equal opportunities?  Do the leaders share updated policies with their workers? |
|  |  | Hand over policy^m^ | | | There is no hand over policy in the department. | | | The leaders are aware of the importance of the hand over policy, but they are not aware of its importance in enhancing knowledge sharing. | | | HCPs are aware of the importance of hand over policy, and the managers have a clear plan to introduce it to the workers. | | | Leaders introduced the hand over policy and endorsed it among health care workers. This policy should cover the willingness to share their knowledge and the knowledge that is gained during work to others to enhance knowledge-sharing process. In addition, anyone who wants to leave departments has a role in handing over responsibility and their knowledge to others to keep the knowledge circulating in the departments. | | | HCPs are fully understanding hand over policy, and they are willing to share any knowledge they have to cover their duties when they are absent. |
|  |  | Measurement | | | Is there a hand over policy during their work and once they leave the department? | | | Has level 1 achieved?  Are the leaders aware of the hand over policy for enhancing knowledge-sharing practices? | | | Has levels 1, and 2 achieved?  Do the leaders have a clear policy for the hand over during their work and once they decide to leave the department? | | | Has levels 1, 2, and 3 achieved?  Are HCPs aware of hand over policies? | | | Has levels 1, 2, 3, and 4 achieved?  Are HCPs willing to share their knowledge during their work and once they decide to leave? |
|  | **Achieving departmental tasks** | | | | | | | | | | | | | | | | |
|  |  | Creating teamwork^n^ | | | There is no teamwork in the medical imaging department. | | | Leaders and HCPs are aware of the importance of teamwork in enhancing knowledge sharing, resulting in an increased number of tasks; however, there is no actual teamwork within the department.  Leaders are aware that diversity in teamwork has several benefits and challenges.  Leaders are aware of the diversity of teamwork that help them to engage with each other based on their background and experience; therefore, enhancing knowledge sharing. However, there are few teamwork in the department. | | | In the medical imaging department. The HCPs work in teamwork, who are a group of professionals who are working together, and sharing their skills, and experiences to perform institutional tasks. Each teamwork has a responsibility to perform the specific tasks in an accurate manner. In addition, there are several teamwork in the department. | | | The departmental tasks are performed by working in teams. In addition, leaders divide HCPs into several teams, each them has a responsibility to perform a specific task based on their experience and skills. The diversity helps in accelerating tasks in the departments. | | | Leaders and health care professionals fully understand the importance of working within team to increase their ability to share their knowledge and increase the number of tasks within the department because each of them is responsible for achieving specific tasks within the group based on their experience and skills. |
|  |  | Measurement | | | Are HCPs working as a group or team? | | | Has level 1 achieved?  Are leaders and HCPs aware of the importance of teamwork, and the diversity of the teamwork itself and other teamwork as a unit within the department? | | | Has levels 1, and 2 achieved?  Are there groups of HCPs working together to perform specific tasks? | | | Has levels 1, 2, and 3 achieved?  Did the leader divide HCPs into several teams to perform specific tasks based on their experience?  Is there a diversity in teamwork in departments? | | | Has levels 1, 2, 3, and 4 achieved?  How fast and accurate the departmental tasks are achieved in the department?  Is there specific teamwork for specific task?  How many teamwork groups are involved in the department, and what are they? |
|  | **Continuous education and developing HCP skills** | | | | | | | | | | | | | | | | |
|  |  | Organizing (learning lectures, workshops, training sessions, physician rounds, and participation in conferences)^o^ | | | There are no continuing education activities. | | | Leaders are aware of the importance of continuing education activities, but they do have a plan or policy to offer these activities to HCPs. | | | Leaders are aware of the importance of continuing education in enhancing knowledge-sharing behaviors by developing their skills in participation in those activities (learning lectures, workshops, training sessions, physician rounds, and participation in conferences, and after that, they acquire new knowledge and want to share it with others. | | | Leaders organize several activities to develop their skills. In addition, they set clear plans and policies to give everyone the chance to participate in those activities and share what they have learned with their colleagues.  Leaders organizepersonalized learning activity for HCPs who need to practice more in specific area.  Leaders in other departments arrange exchange expertise among each other as a part of continues learning. | | | Leaders fully understand the impotence of continuing education. In addition, there is a clear policy that encourages workers to participate in those activities and allows them after that to share their knowledge with others to maximize the benefits from those activities. In addition, they are fully aware of the importance of the personalized training activities based on HCPs need. |
|  |  | Measurement | | | Are there continuing educational activities in the department? | | | Has level 1 achieved?  Are leaders aware of the importance of continued education in enhancing knowledge sharing? | | | Has levels 1, and 2 achieved?  Are leaders using continuous education to develop HCPs skills? | | | Has levels 1, 2, and 3 achieved?  Is there a clear policy to manage participation in continuous educational activities?  Are there personalized learning activities for HCPs based on their need?  Are there clear policies to exchange expertise among other departments? | | | Has levels 1, 2, 3, and 4 achieved?  What are the activities that the department offers to HCPs? |
|  | **Making decision** | | | | | | | | | | | | | | | | |
|  |  | Regular meeting^p^ | | | There are no regular meetings either formal or informal in the department.  Most of the HCPs do not know about any updates in the department. | | | Leaders are aware of the importance of regular meetings in informing them of any updates in the department, but there is no regular meetings among them.  Leaders are aware of the importance of informal meetings in creating cultural environment rich of knowledge sharing. | | | Any updates that are introduced in the department, leaders organize meetings to discuss them with workers to share knowledge, ideas, and allow them to participate in anything that is introduced in the department  For any special occasions, there are informal meetings among HCPs that help to increase knowledge-sharing behaviors. | | | In the department, there are several meetings that happen regularly, such as morning or weekly or monthly meetings, and each meeting has a specific purpose, such as making decision, creating, new protocol, solving problems, and discussing incident reports. In addition, there are informal meeting that are related to any social activity, such as (new grade, having baby, national day, and so on) that allow them to engage with each other, and therefore, increase knowledge-sharing practices. | | | HCPs are fully aware of the purpose of those meetings, and they are regularly involved in them. In addition, for those who are not able to attend those meetings, there are follow-up meetings. |
|  |  | Measurement | | | Is there a regular meeting (formal or informal) in the department? | | | Has level 1 achieved?  Are leaders aware of the importance of the meetings (formal or informal) in enhancing knowledge sharing? | | | Has levels 1, and 2 achieved?  Have leaders organized meetings to inform HCPs about any updates in the department?  Have leaders organized informal meeting related to their achievements to increase the level of engagement? | | | Has levels 1, 2, and 3 achieved?  Is there a regular policy for setting those meetings in the department? | | | Has levels 1, 2, 3, and 4 achieved?  Is the percentage of attendance in those meeting high?  Is there a follow-up meeting for those who cannot attend? |
|  |  | MDT^q^ and CoP^r^ making decision^s^ | | | Not all health care professionals know about those professional meetings. | | | Leaders are aware of the importance of those meetings in making decisions, but most HCPs are not aware of or know about them, and they are not involved in those meetings. | | | In the medical imaging departments, health care professionals participate in several specialized meetings, such as MDT, CoP, and CoO^t^. Those meetings contain different workers from different disciplines, such as: radiology, radiographers, nurses, oncologists, surgeons, and radiotherapists to share their knowledge, and therefore, enhance making decisions based on the specific cases. | | | In the department, there are several specialized meetings, each of those meetings is specialized in certain diseases, and all HCPs are involved in those meetings routinely to share their knowledge and create new one.  HCPs participate in those meetings for training purpose.  Leaders are aware about who are involved in those specialized meetings to avoid wasting their time. | | | All HCPs are fully aware of those meetings, and all of them have a chance to participate in those meetings if they are related to them. In addition, the representative in each field can attend those meetings in other hospitals and share the outcomes to others who are interested. |
|  |  | Measurement | | | Are there specialized meetings in the department? | | | Has level 1 achieved?  Are leaders aware of the importance of the specialized meetings in the department? | | | Has levels 1, and 2 achieved?  Are HCPs participating in the specialized meeting to make decisions based on the specific cases? | | | Has levels 1, 2, and 3 achieved?  Is there a clear policy to manage who are attending specialized meetings? | | | Has levels 1, 2, 3, and 4 achieved?  Are there representative members to attend those meetings in another hospital? |
|  | **Infrastructure and workforce** | | | | | | | | | | | | | | | | |
|  |  | Meeting room and office layout^u^ | | | In the medical imaging department, there is no room for sharing knowledge, or offices for HCPs. | | | Leaders are aware of the importance of spaces to share their knowledge, but there are not enough spaces to practice that, or available offices to all HCPs. | | | There is enough room in the department that has a significant role in performing circular discussions, and formal meetings.  For emergency cases or as an alternative space, there is an online space to allow them to meet and share their knowledge  There are enough offices for all HCPs. | | | In the department, there is a specific schedule for the periodical meetings among HCPs with their leaders. In addition, there are enough offices for employees to perform their tasks.  For the informal meeting, there is a coffee station in that space for gathering that helps them to share their knowledge. | | | Leaders fully understand the importance of spaces, and office layout to enhance knowledge-sharing practices among health care workers. In addition, any HCP has the opportunity to use the room to meet others either formal or informal. |
|  |  | Measurement | | | Is there room for knowledge sharing in the department? | | | Has level 1 achieved?  Are leaders aware of the importance of space in enhancing knowledge sharing? | | | Has levels 1, and 2 achieved?  Are there enough rooms and offices for all HCPs to share their knowledge?  Is there an online room for meetings as an alternative space? | | | Has levels 1, 2, and 3 achieved?  Is there a specific schedule for performing formal meetings in those spaces?  Is there a coffee corner for the informal meeting space? | | | Has levels 1, 2, 3, and 4 achieved?  Are the offices fit for all HCPs? |
|  |  | Enhanced extrinsic motivation^v^ | | | There is no extrinsic motivation in the department. | | | Managers are aware of the importance of extrinsic motivation in enhancing knowledge sharing, but there is a lack of policy for extrinsic motivation. | | | In the department there are several extrinsic motivation activities that help HCPs practice knowledge sharing to receive those incentives. | | | In the department, there are several incentives (giving bones and thanking certificate) | | | Managers fully understand the importance of setting clear policies for incentives to maximize the level of sharing knowledge. |
|  |  | Measurement | | | Are there incentives in the department? | | | Has level 1 achieved?  Are managers aware of the importance of extrinsic motivation in enhancing knowledge sharing? | | | Has levels 1, and 2 achieved?  Is there a variety of extrinsic motivation in the department? | | | Has levels 1, 2, and 3 achieved?  Are there physical and emotional motivation in the department? | | | Has levels 1, 2, 3, and 4 achieved?  Is there clear policy for extrinsic motivation in the department? |
|  |  | Organized work process^w^ | | | There is no clear plan to organize the workflow process. | | | Leaders are aware of the importance of an organized work process, but there is no clear plan to organize the workflow process in the department. | | | Leaders have a clear plan for how to organize activities among HCPs. In addition, they are grouping this activity into work units to increase the number of performing tasks. | | | The workflow process is divided among HCPs, each of them have a specific job to perform tasks. Therefore, all HCPs know how to perform tasks in departments. In addition, time is available to practice knowledge-sharing activities among them by attending meetings and continuing education practices (mentioned in indicator 9). | | | Leaders fully understand the importance of an organized workflow process, and there is a clear plan to perform the tasks within the department. In addition, all HCPs are aware of how to perform the tasks within their department, and they can cover for others in their absence.  There is plenty of time due to the organized workflow process to allow them to practice knowledge-sharing activities. |
|  |  | The measurement | | | Is there clear plan for the workflow process? | | | Has level 1 achieved?  Are leaders aware of the importance of organized work in enhancing knowledge sharing? | | | Has levels 1, and 2 achieved?  Are HCPs working based on an organized plan that is set monthly? | | | Has levels 1, 2, and 3 achieved?  Are workflow and departmental tasks divided among HCPs? | | | Has levels 1, 2, 3, and 4 achieved?  Is there a clear policy on how to organize workflow?  Is there enough time to allow HCPs to participate in knowledge-sharing activities? |
| **Technological factors^x^** | | | | | | | | | | | | | | | | | |
|  | **Stored and shared patient data electronically**. | | | | | | | | | | | | | | | | |
|  |  | Strong network^y^ | | | There is no access to the network in the department. | | | Leaders are aware of the importance of networks in the department in sending and receiving data electronically, and therefore enhancing knowledge-sharing activities.  Leaders are aware of the importance of strong network in adopting new artificial intelligence technologies that help in increasing knowledge-sharing practices; therefore, increasing patient’s outcome, and reducing medical errors. | | | There is a strong network in the department, helping in sending and receiving the data among other departments in the local hospital or around the country, and adopting new artificial intelligence technologies. | | | On the basis of the strong network infrastructure, there are several technological modalities introduced in the department, such as HIS^z^, RIS^aa^, and PACS^ab^. In addition, it allows the use of social media platforms to share knowledge among workers electronically, such as, WhatsApp and Zoom.  There are several artificial intelligence technologies in department that help the professionals to speed up performing tasks. | | | There is a strong network that allows leaders to use electronic tools to practice knowledge sharing among workers. In addition, there is a clear policy to update and maintain the network in the department.  Leaders are fully aware of the importance of the network in routine work. |
|  |  | Measurement | | | Is there access to the network in the department? | | | Has level 1 achieved?  Are leaders aware of the importance of networks in enhancing knowledge sharing? | | | Has levels 1, and 2 achieved?  Is there a strong network in the department to send and receive patient files, and interpret accurate data? | | | Has levels 1, 2, and 3 achieved?  Are there ICT^ac^ infrastructures available in the department?  Are there several technologies obtained in department? | | | Has levels 1, 2, 3, and 4 achieved?  Is there a clear policy to obtain a high-speed network and maintain it in the department? |
|  |  | Implementation of ICT and maintenance^ad^ | | | There is no implementation of ICT infrastructure. | | | Leaders are aware of the importance of ICT. However, not all HCPs know how to use it efficiently. | | | ICT infrastructure plays an important role in enhancing knowledge sharing by speeding up that transfer process of the data that is needed to perform specific tasks, such as interpreting results and making decisions. | | | In the department, there are a variety of ICT infrastructures. Each of them has a specific purpose for using it. For example:  HIS, RIS, and PACS: uploading and downloading patient data, history, scans, and laboratory reports to speed up.  Intranet or Extranet: for departmental circulars, and policies, manuals, and protocols.  Social media platforms: WhatsApp, Zoom, Teams, Facebook, and so on, for sharing knowledge within the department, and using them in an emergency case to establish meetings electronically.  HCPs attend workshops to learn how to deal with this technology. | | | The departments are fully using ICT infrastructure. In addition, leaders and HCPs need to be aware of how to deal with this technology properly.  There is periodically maintenance, and a clear policy to develop, and update this technology. |
|  |  | Measurement | | | Are there implementations of ICT infrastructures in the department? | | | Has level 1 achieved?  Are leaders aware of the importance of ICT in enhancing knowledge sharing? | | | Has levels 1, and 2 achieved?  Does ICT have a role in speeding up the process of performing the departmental tasks? | | | Has levels 1, 2, and 3 achieved?  Are HCPs trained well enough to allow them to use these infrastructures properly?  Are there a variety of ICT modalities in the department? | | | Has levels 1, 2, 3, and 4 achieved?  Is there a clear policy for periodical maintenance of ICT infrastructure?  Is there a clear policy for the use of ICT tools? |
|  | **Access to the electronical databases** | | | | | | | | | | | | | | | | |
|  |  | Digital resources^ae^ | | | There is no access to the electronic databases. | | | Leaders are aware of the importance of digital resources, but there is no access to the electronic databases. | | | HCPs are able to access electronic databases to read and gain knowledge based on other works that have been publicized through a medical journal. However, there is limited access to those databases, such as, for physicians only. | | | In the department, there are digital libraries that allow HCPs to access several international journals to read, and therefore to discuss them with other peers. | | | The department has fully implemented electronic databases. In addition, there is a digital library in the department, and all health care workers are eligible for access to it. |
|  |  | Measurement | | | Is there digital resources in the department? | | | Has level 1 achieved?  Are leaders aware of the importance of digital resources? | | | Has levels 1, and 2 achieved?  Are HCPs able to access the electronic databases?  Are all HCPs granted permission to access those databases? | | | Has levels 1, 2, and 3 achieved?  In the department, are there journal clubs to discuss the recent articles? | | | Has levels 1, 2, 3, and 4 achieved?  Is the department fully implemented in the digital library, resources, and do all HCPs have permission to access it? |

^a^It is the first phase of introducing knowledge-sharing practices among health care professionals. It measures by how fast knowledge reach health care professionals within department and how fast the tasks were performed in an efficient way.

^b^KS: knowledge sharing.

^c^Indicator 1/17: the health care professionals in the medical imaging department are aware of the importance of knowledge-sharing behaviors, facilitators, and the factors that affect knowledge-sharing practices.

^d^HCPs: health care professionals.

**^e^**It consists of any data, or information that indicates a certain amount of knowledge either tacit or explicit knowledge, needing to share it to create new knowledge.

^f^Indicator 2/17: HCPs are aware of both types of knowledge and how it is structured, and collected to enhance knowledge-sharing practices

^g^It consists of several individual characteristics that play an important role in sharing their knowledge. It is the key to successful knowledge-sharing practices because it relies on the people.

^h^Indicator 3/17: Budling trust among HCPs and their managers helping to circulate knowledge sharing among them, therefore increasing number of tasks.

^i^Indicator 4/17: intrinsic motivation is one of the important individual facilitators that allow knowledge-sharing behaviors circulating among HCPs

^j^Indicator 5/17: the personality and positive attitudes of HCPs are directedly related to the good communication among them; therefore they enhance knowledge sharing

^k^It is the ability of the departments to achieve institutional tacks, increased patient outcome by enhancing knowledge-sharing practices among HCPs

^l^Indicator 6/17: Head of departments, and senior managers are the leader in their department. They have a responsibility to build cultural knowledge sharing environments to enhance knowledge-sharing practices.

^m^Indicator 7/17: hand over policy: it is the policy that helps to keep the knowledge either tacit or explicit circulating among HCPs, and it has positive impact on increasing knowledge-sharing practices.

^n^Indicator 8/17: Creating teamwork within workplace is important to achieve tasks, and procedures in an efficient manner by enhancing knowledge sharing among HCPs professionals.

^o^Indicator 9/17: organizing continuous education activities, such as: learning lectures, workshops, training sessions, physician rounds, and participation in conferences helps in developing HCPs’ skills by enhancing knowledge-sharing activities among them

^p^Indicator 10/17: regular meetings either between leaders and HCPs, or among HCPs are one of the important factors that increase knowledge-sharing activities, and therefore increase patients’ outcomes

^q^MDT: multidisciplinary team.

^r^CoP: communities of practice

^s^Indicator 11/17: The specialized meetings, such as MDT and CoP have a significant role in enhancing sharing knowledge by setting clear treatment plan based on patient case. There are several professionals from different specialized fields who are involved in those meetings.

^t^CoO: community of oncologists

^u^Indicator 12/17: the medical imaging department has an ~~empty~~ room for meetings, and there is an organized layout that enhances knowledge-sharing practices

^v^Indicator 13/14: extrinsic motivation is of the important factors that enhances knowledge sharing among HCPs. It could be physically or emotionally.

^w^Indicator 14/17: organized work process is one of the important indicators that enhances knowledge sharing by giving them clear plans to achieve work processes, enough time, and fair opportunities to practice knowledge-sharing activities

^x^It consists of using both types of technology infrastructure: hardware and software that allow HCPs to perform their tasks efficiently

^y^Indicator 15/17: Strong network plays a vital role in enhancing knowledge sharing by introducing new technology to store, and share the data, anytime and anywhere

^z^HIS: Hospital Information System

^aa^RIS: Registration Information System

^ab^PACS: picture archiving and communication system

^ac^ICT: information and communication technology

^ad^Indicator 16/17: implanting ICT infrastructure, such as: PACS, social media, intranet, extranet, telemedicine, teleradiology play very important role in enhancing knowledge-sharing behaviors.

^ae^Indicator 17/17: digital resources are one of the important technological facilitators that enhance knowledge by developing HCP’s knowledge through accessing the updated articles and journals.
