# Supplementary material for: Knowledge Sharing Maturity Model for Medical Imaging Departments: Development Study
Source: JMIR Hum Factors. 2025 May 6;12:e54484. doi: 10.2196/54484 (PMC12093077; doi:10.2196/54484)
Supplement: Multimedia Appendix 5 [file humanfactors_v12i1e54484_app5.docx]

| Multimedia Appendix 5: The results of the Delphi methods in two rounds. | | | | | | |
| --- | --- | --- | --- | --- | --- | --- |
| Indicator | Results (R1) | | | Results (R2) | | |
|  | Mean (3.4-5) | ICC>66% | SD>1 | Mean | ICC | SD |
| Awareness | | | | | | |
| Awareness | | | | | | |
| 1. Awareness and willingness towards KS | | | | | | |
| (1/17): The healthcare professionals in the medical imaging department are aware of importance of knowledge sharing behaviours, and facilitators and the factors that affect knowledge sharing practices. | 4 | 66.6% | 1.2 | 4.4 | 88.8% |  |
| Types of knowledge sharing | | | | | | |
| Understanding and organizing knowledge sharing repository | | | | | | |
| 1. Structured, and collected both types of knowledge | | | | | | |
| (2/17): Healthcare professionals aware of both types of knowledge and how its structured and collected to enhance knowledge sharing practices. | 4.3 | 100% | 0.4 | 4.2 | 77.7% |  |
| Individual factors | | | | | | |
| Communication among healthcare professionals. | | | | | | |
| 1. Building trust among healthcare professionals, and share their experience | | | | | | |
| (3/17): Budling trust among healthcare professionals and their managers helping to circulate knowledge sharing among them, therefore increasing number of tasks. | 4 | 77.7% | 0.9 | 4.4 | 88.8% | 0.68 |
| 1. Increased intrinsic motivation (self-efficacy, and self-esteem) | | | | | | |
| (4/17): Indicator 4/17: Intrinsic motivation is one of the important individual facilitators that allow knowledge sharing behaviours circulating among healthcare professionals | 4.1 | 88.8% | 0.8 | 4.6 | 88.8% | 1 |
| Personality and positive attitudes | | | | | | |
| 1. Personality and communication among healthcare professionals | | | | | | |
| (5/17): The personality and positive attitudes of healthcare professionals are directedly related to the good communication among them, therefore enhance knowledge sharing | 4.1 | 88.8% | 0.8 | 4.3 | 77.7% | 0.68 |
| Departmental factors | | | | | | |
| Leadership and culture | | | | | | |
| 1. Structured leadership and creating culture. | | | | | | |
| (6/12): Head of departments, and senior managers are the leader in their department. They have responsibility to build a cultural knowledge sharing environments to enhance knowledge sharing practices. | 4.4 | 100% | 0.5 | 4.3 | 77.7% | 0.67 |
| 1. Handover policy | | | | | | |
| (7/12): Handover policy: it is the policy that helps to keep the knowledge either tacit or explicit circulating among healthcare professionals, and it has positive impact on increasing knowledge sharing practices. | 4.3 | 88.8% | 0.9 | 4.4 | 88.8% | 1 |
| Achieving departmental tasks | | | | | | |
| 8. Creating teamwork | | | | | | |
| (8/17): Creating teamwork within workplace is important to achieve, tasks, and procedures in an efficient way by enhancing knowledge sharing among healthcare professionals. | 4.4 | 100% | 0.5 | 4.5 | 88.8% | 0.82 |
| Continuous education and develop HCP skills | | | | | | |
| 1. Organising (learning lectures, workshops, training sessions, doctor rounds, and participation in conferences | | | | | | |
| (9/17): Organising continues education activities such as: learning lectures, workshops, training sessions, doctor rounds, and participation in conferences helps to developing healthcare professionals’ skills, by enhancing knowledge sharing activities among them. | 4.5 | 100% | 0.5 | 4.4 | 88.8% | 0.68 |
| Decision making | | | | | | |
| 1. Regular meeting | | | | | | |
| (10/17): Regular meetings either between leaders and healthcare professionals, or among healthcare professionals are one of important factor that increased knowledge sharing activities, therefore, increased patients’ outcomes. | 4 | 77.7% |  | 4.5 | 77.7% | 1 |
| 1. MDT and CoP for making decision | | | | | | |
| (11/17): The specialized meetings such as MDT, and CoP have a significant role in enhancing sharing knowledge by setting clear treatment plan based on patient case. There are several professionals from different specialised fields are involved in those meetings. | 4 | 77.7% | 0.9 | 4.3 | 88.8% | 0.68 |
| Infrastructure and workforce | | | | | | |
| 1. Meeting room and office layout | | | | | | |
| (12/17): In the medical imaging department has an empty room for meetings, and there is organized layout that enhances knowledge sharing practices | 4 | 77.7% | 0.9 | 4.5 | 88.8% | 0.47 |
| 1. Enhanced extrinsic motivation | | | | | | |
| (13/17): Extrinsic motivation is of the important factors that enhances knowledge sharing among healthcare professionals. It could be physically or emotionally. | 3.6 | 77.7% | 0.6 | 4.4 | 88.8% | 0.69 |
| 1. Organised work process | | | | | | |
| (14/17): Organized work process is one of important indicators that enhances knowledge sharing by giving them clear plan to achieves work process, enough time, and fair opportunities to practice knowledge sharing activities | 4.4 | 88.8% | 0.6 | 4.5 | 88.8% | 0.44 |
| Technological factors | | | | | | |
| Stored and shared patient data electronically | | | | | | |
| 1. Strong network | | | | | | |
| (15/17): Strong network plays vital role in enhancing knowledge sharing by introducing new technology to store, and share the data, anytime and anywhere. | 4.4 | 100% | 0.5 | 4.3 | 77.7% | 0.47 |
| 1. Implementation information, communication technology and maintenance | | | | | | |
| (16/17): Implanting information communication technology infrastructure such as: PACS, social media, intranet, extranet, tele-medicine, teleradiology play very important role in enhancing knowledge sharing behaviours. | 4.2 | 88.8% | 0.9 | 4.7 | 88.8% | 0.67 |
| Access to the electronical databases | | | | | | |
| 1. Digital Libraries | | | | | | |
| (17/17): Digital libraries are one of important technological facilitators that enhance knowledge by developing healthcare professional’s knowledge through accessing to the updated articles and journals. | 4 | 77.7% | 1.1 | 4.5 | 88.8% | 0.4 |
