# Supplementary material for: Knowledge Sharing Maturity Model for Medical Imaging Departments: Development Study
Source: JMIR Hum Factors. 2025 May 6;12:e54484. doi: 10.2196/54484 (PMC12093077; doi:10.2196/54484)
Supplement: Multimedia Appendix 6 [file humanfactors_v12i1e54484_app6.docx]

**The comments**

(1/17):

(R7) I would separate "Awareness" from "willingness". they are very different concepts and have to be measured differently. I noticed the explanation and measurements are focused on awareness more than willingness s.

Thank you for your comment. That’s right. Additionally, based on the Systematic Review the awareness is considered as facilitators in the medical imaging departments not the willingness. Therefore, I will keep the awareness only as main indicators.

(R5) Its apart of our job to share knowledge between health workers to increase skills.

Thanks for your comment. Yes, it is. I Agee with you, and that is main outcome of the knowledge sharing is to increase their skills.

(2/17):

(R7) Optimizing the sharing of tacit knowledge is very different than optimizing the sharing of explicit knowledge! Having a policy in place can only optimize the explicit sharing! the sharing of tacit is very much internally triggered and motivated! external policy can be limited in it's effect.

Thank you for your comment. The change has done based on the optimising the explicit knowledge happens through having and setting clear policies that help them to reach those documents that explicitly documented. On the other hand, the optimizing the tacit knowledge can be done through externalization by converting the tacit knowledge to the explicit one by commentating the important talk on the meetings as notes to summary the meeting or for those who were not attending.

(R6) Practitioners may make use of the two forms of knowledge without being explicitly aware of or being able to articulate a discriminatory between the two.

Thank you for your comments, yes, that right, and there is a huge responsibility from the managers to explain the difference between them and how to structure them.

(R5) There is no policy to share awareness, but there is evaluation to all workers and responsibility to share knowledge between them.

Thank you for your comment. There is policy for knowledge sharing, and that is main responsibility for the manager due to the important role of knowledge sharing practices among healthcare professionals.

(R4) Probably terms are new. Tacit knowledge may be shared but not received easily by others.

Thank you for your comment, yes, that right, therefore, we need to externalization process that help to convert the tacit knowledge to the explicit one by documenting the meeting talk as main points and share it with others.

(R1)I don't think it's up to us to teach healthcare professionals what good data looks like. They're highly trained, they should know.

Thank you for your comment. They are well qualified in their professionals. However, the have to know how the know about the types of the knowledge that existing in the area that they work on it to increase knowledge sharing practices therefore increasing patients’ outcomes.

(3/17):

(R7) there are two kinds of trust that has to be addressed " trust in the competencies and expertise of the other" and "benevolence trust" and these should be measured separately. For 4-17 I would definitely separate in measurement between self-efficacy and self-esteem.

Thank you for your comment. The changes have been done. I separate the two types of trust, each of them has a role in enhancing knowledge sharing practices. The trust in their abilities and other expertise help to perform more tasking in working area, and the benevolence trust help to reduce the conflicts among them, therefore increasing knowledge sharing.

(R6) Perhaps there should be more distinction between being aware of the importance of trust and there being trust. What of the case where trust was not considered important to knowledge sharing but there was a high degree of trust amongst colleagues.

Thank you for your comment. I did not agree with you. Trust is one of important facilitator that affect on knowledge sharing practices by increasing the achieved tasks because they are trust their abilities and other expertise’s.

(R5) Because we are in a Cancer Control Center for sure, trust is important.

Thank you for your comment, yes, I agree with you.

(R1) Often knowledge sharing is limited by factors external to the knowledge-sharing process, such as understaffing and under-funding causing stress.

Thank you for your comment, yes, am totally agree with you that shortness of the staff and under-funding will affect on knowledge sharing practices. However, the knowledge sharing practices is not limited to external factors, we want them internally to feel confident and trust of their abilities and other expertise to share their knowledge.

(4/17):

(R7) see 3-17- not to mix between self-efficacy and self-esteem in measurement.

Thank you for your comment, changes have done, look at the indicator (4/17). And yes, they are different in how managers help their professionals to seek optimal challenges, and they have to be aware of it, which directly related to the intrinsic motivation.

(R1)Yes, people need to be motivated but managers need to set departmental and individual priorities and create capacity for CPD.

Thank you for your comment, Yes am agreed with you.

(5/17):

(R7) personality and positive attitude are linked to internal factors as well as external factors! so the communication between health care professionals can be a result of the personality types too!

Thank you for your comment. Yes, it is and both of them are part of the individual facilitators that helps in enhancing knowledge sharing practices, and therefore good communication among them. So that, managers should be aware of the types of personality and respect them for best communication.

(R5) Sometimes there is a conflict, but it can be managed because its chief technologist responsibility to guid, improve and develops responsibilities.

Thank you for your comments. Yes, that’s right.

(6:17):

(R7)I would also add "are the leaders modelling a knowledge sharing behaviour in their departments?" before providing a space only!

Thank you for your comment. Yes, I add it in the first level, they have to aware of the knowledge sharing models and how they create one based on available resources.

(R6) Level one seems unlikely to ever occur though.

Thank you for your comment. Yes, it is. I have removed it and added instead of it (The managers or senior departments do not have enough knowledge about modelling knowledge sharing to create a culture for communication in their department). That is very important for any leader.

(R5) There is a policy for all workers and a work plan as well as all workers updated with the police.

Thank you for your comment. That is really good to have a policy to follow it.

(R1) As HoD, you are responsible for everything, like it or not!

Thank you for your comment. Yes, it is. But they have to be aware in how they apply it adopt it.

(7/12):

(R7) You need to make sure in the questions that you are covering the willingness to share "while" they are there also and not only when they decide to leave!

Thank you for your comment. Yes, that right. I have not aware of it. I updated the indicator.

(R5)There is a handover policy all workers are aware of it.

Thank you for your comment. That is great to hear that you have it and the aware of it.

(8/17):

(R7) a need to cover "diversity" in teamwork.

Thank you for your comment. I have mentioned that in general, but I added more details about the diversity and how its important in the department to increase the performing task in the department.

(R5) Our work is imaging, all workers do study as required it is not fast task it is accurate procedure. All the team distributed to cover cases in camera and other site. We have 20 Technologists.

Thank you for your comment. That’s sounds good.

(9/17):

(R7) a need to integrate the "personalized" approach to learning in the questions!

Thank you for your comment. I added the personalised learning for the HCPs based on their need. Additionally, the exchange the experts among departments as part of the learning also, therefore, increase knowledge sharing practices.

(R5) In Nuclear Medicine there is continues education activities for doctors, technologists, and nurses. Meetings, lectures, and conferences

Thank you for your comment. That sounds good that you are applying this at your department.

(10/17):

(R7) you are missing questions around "informal" meetings? where knowledge sharing can be much richer

Thank you for your comment. Yes, that right I missed it. I added those meeting in this indicator as part of the regular meeting that increases the level of engagements.

(R6) There are many types of meeting some important some not. Also, the outcome of meetings and whether they have an impact is perhaps more important.

Thank you for your comment. Yes, it is. There are formal and informal meeting and both of them have an important role in knowledge sharing.

(R5) Meetings are on a regular schedule.

Thank you for you comment. That sounds good. But you have to be aware of different types of meetings.

(R1) People understanding the purpose of meetings isn't just a departmental culture thing, it's down to individual engagement too.

Thank you for your comment. Yes, it is. Therefore, I added the informal meetings that help to increase the level of the engagement among them.

(11/17):

(R5)Yes, all the workers can attend the meetings.

Thank you for your comment. That sound good, but you have to be aware of what is related to them or not.

(R1) Not all meetings are suitable for all team members, and you shouldn't waste people's time if they're not.

Thank you for this comment. That is a good point. I added to the model to be aware of it.

(12/17):

(R7) Having the water cooler concept or a coffee station is also a space where informal meetings can take place.

Thank you for your comment. Yes, I agree with you I added it.

(R6) I am not convinced that the existence of ‘empty space’ is necessarily important or inducive to knowledge sharing.

Thank you for your comment. Yes, its not. But it should be a space. Therefore, I excluded the world empty.

(R5) Departmental meeting done in hospital meeting room which fit for all workers and meeting to discuss causes done in reporting room which is fit to display cases.

Thankyou for your comment. Yes, I agree with you. Additionally, it should be convenient to fit all professionals.

(R1) Knowledge sharing is not dependent on empty spaces, and the many teams who operate purely online will testify.

Thank you for this comment. Yes, I am agreeing with you. So that, I added online space as an alternative space for meetings and in an emergency case.

(13/7):

(R7) the question in level 4 covers "emotional" which is intrinsic and needs to be deleted.

Thank you for your comment. Yes, I deleted.

(R1)The NHS doesn't offer incentives in this way - no bonuses or prizes.

Thank you for your comment. That’s really disappointed but giving them thankful certificate will be a part of incentives that cost nothing.

(14/17):

(R7) I am not sure of the extent to which workflow organisation necessarily leads to knowledge sharing.

Thank you for your comment. Organising workflow is important to facilitate knowledge sharing practices by giving the healthcare professionals a clear plan for each month and informing them what are their responsibility. That organising will help them to organise between work and attending activity in the department by give everyone a time and chance to participate in knowledge sharing activity.

(R6) Yes, there is a clear policy for all workers to follow work follow and do theirs task, and they have enough time to share knowledge activity

Thank you for your comment. That sounds good.

(15/17):

(R7) I would include questions about "AI" and its role and not only "networks

Thank you for your comment. I meant from the Strong network is using strong WIFI network that allow professionals to use it in adopting several technologies. I added AI as one of the several technologies in the department that need strong network to run it.

(R5) Since we are connected to a health information system, an updating is an amust, and we have different system Pasricha’s with high speed network

Thank you for your comment. That sounds good.

(R1) Network also needed for routine work.

Thank you for your comment. Yes, I am agreeing with you.

(16/17):

(R7) I would ask in level 5: is there a clear policy for the use of ICT tools? [the do's and don’ts]

Thank you for your comment. That really important. Sure, I added it as question in L5.

(R5)Yes, there is a policy to update all the system.

Thank you for your comment. That’s sound good.

(R1) NHS budget does not support modern ICT.

Thank you for your comment. That is really disappointed. But if there were enough ICT in a good condition it will save time and allow more tasks to be performed.

(17/17):

(R7) I believe you are missing on many other digital resources that can be accessed through subscriptions and can be more valuable that having only a digital library! having a portal that can be a gate to many other external electronic resources is very important also.

Thank you for comment. I have signposted to the databases as gate for many resources. Therefore, I changed the indicator to the digital resources to focus on the databases in general.

(R5) Yes, the department works with a digital system, and everyone can access.

Thank you for your comment. Accessing to the network and digital system is different than accessing to the databases that required subscription to allow the professionals to access the professional databases.

(R1) Again, not set up due to budget and staffing limitations.

Thank you for your comment. That is really disappointed and should a clear policy for it.
